# Alzheimer’s Disease—Rationales for Potential Treatment with the Thrombin Inhibitor Dabigatran

**DOI:** 10.3390/ijms22094805

**Published:** 2021-04-30

**Authors:** Klaus Grossmann

**Affiliations:** Center for Plant Molecular Biology (ZMBP), University of Tübingen, 72076 Tübingen, Germany; Klaus.grossmann@uni-tuebingen.de

**Keywords:** Alzheimer’s disease, brain amyloidosis, cerebral amyloid angiopathy, vascular dysfunction, amyloid-β-proteins, thrombin, fibrin, thrombin inhibition, direct oral anticoagulant, dabigatran

## Abstract

Alzheimer’s disease (AD) is caused by neurodegenerative, but also vascular and hemostatic changes in the brain. The oral thrombin inhibitor dabigatran, which has been used for over a decade in preventing thromboembolism and has a well-known pharmacokinetic, safety and antidote profile, can be an option to treat vascular dysfunction in early AD, a condition known as cerebral amyloid angiopathy (CAA). Recent results have revealed that amyloid-β proteins (Aβ), thrombin and fibrin play a crucial role in triggering vascular and parenchymal brain abnormalities in CAA. Dabigatran blocks soluble thrombin, thrombin-mediated formation of fibrin and Aβ-containing fibrin clots. These clots are deposited in brain parenchyma and blood vessels in areas of CAA. Fibrin-Aβ deposition causes microvascular constriction, occlusion and hemorrhage, leading to vascular and blood–brain barrier dysfunction. As a result, blood flow, perfusion and oxygen and nutrient supply are chronically reduced, mainly in hippocampal and neocortical brain areas. Dabigatran has the potential to preserve perfusion and oxygen delivery to the brain, and to prevent parenchymal Aβ-, thrombin- and fibrin-triggered inflammatory and neurodegenerative processes, leading to synapse and neuron death, and cognitive decline. Beneficial effects of dabigatran on CAA and AD have recently been shown in preclinical studies and in retrospective observer studies on patients. Therefore, clinical studies are warranted, in order to possibly expand dabigatran approval for repositioning for AD treatment.

## 1. Introduction

In Alzheimer’s disease (AD), neurodegenerative but also contributing vascular and hemostatic changes in the brain lead to the loss of memory and cognitive skills, robbing humans ultimately of their essential personality. To date, there is no medicine in clinical use or recognizable in the research pipeline that can effectively combat AD, a disease, which affects more than 1 million people in Germany and 40 million worldwide [1,2,3,4,5,6]. Of these individuals, less than 10% develop symptoms well before the age of 65 due to their hereditary predisposition, known as early-onset AD [4].

Currently, the drugs, which can be used for treatment of AD, are only able to dampen dementia symptoms and delay progression for a certain period of time [5]. To these standard AD therapeutics belong cholinesterase inhibitors, such as donepezil, galantamine, rivastigmin, and glutamate antagonists like memantine [5]. Therefore, an urgent task of pharmaceutical research is to search for novel, more effective drugs to possibly cure or considerably mitigate AD [3,4]. Another strategy is to look for new therapeutic approaches, which are based on already known active ingredients with other approval for use [6].

Beginning already in the 1970s, clinical studies with small cohorts of patients with senile dementia revealed a beneficial effect on the disease when treated with the anticoagulative vitamin K antagonist (VKA) warfarin [7,8,9]. One study even dates back to the 1960s and found that morbidity and mortality of dementia patients could be reduced by 50% with treatment of the VKA dicumarol [10]. Dicumarol was originally isolated from plant sweet clover hay [11]. It was introduced into clinical medicine in 1941, as the first antithrombotic drug for patients with cardiovascular disease [11]. Recent results on the contribution of cerebrovascular and hemostatic dysfunction to AD have brought the idea back into focus that anticoagulants may also be beneficial for treatment of AD. As key drivers in vascular pathogenesis and derived neurodegenerative changes, progressive accumulation of toxic amyloid-β proteins (Aβ), thrombin and fibrin have recently been identified that can be treated by anticoagulants [12,13]. In this review article, why the orally active thrombin inhibitor dabigatran could be useful in therapy for cerebrovascular and related cognitive dysfunction in AD is discussed.

## 2. Hemostasis—Anticoagulants Inhibit Thrombin and Fibrin Formation

The multi-stage process of hemostasis prevents excessive blood loss in the organism in the case of injuries to the blood vessel system [14]. In the process of blood clotting (coagulation), a soluble protein from the blood, fibrinogen, is converted into insoluble fibrin, in order to close and heal a wound. Fibrin forms a fiber network with integrated erythrocytes and platelets, a fibrin clot (thrombus). The production of the responsible enzyme, thrombin, which is released from its precursor protein prothrombin, is regulated in a cascade by a variety of tissue and coagulation factors, such as factor Xa. Some of these are activated beforehand in a vitamin K-dependent process [14]. On the other hand, the proteolytic enzyme plasmin degrades fibrin in the fibrinolysis process and thus dissolves fibrin clots [14]. Blood clotting is stimulated and, concomitantly, the risk of formation of harmful thrombi increases, particularly in the case of hereditary modified coagulation factors, large wounds after surgery and injuries, and slower blood flow through incidents, such as atrial fibrillation, damaged blood vessels due to atherosclerosis, and limited physical exercise. These thrombi can trigger occlusion of blood vessels in the process of thrombosis or, after detachment, thrombi move through the vascular system to organs and can cause, e.g., pulmonary embolism or brain infarction [14].

To avoid occurrence of thromboembolism, anticoagulants are used, prophylactically or therapeutically, to inhibit blood clotting. For acute treatment of venous thrombosis and thrombosis prophylaxis in risk situations, such as after surgery, short-term anticoagulation is indicated. Persistent anticoagulation is prescribed, e.g., for the prophylaxis of thromboembolism in patients with cardiac arrhythmias, such as atrial fibrillation, increased cardiovascular risk, or mechanical heart valve replacement [15]. For antithrombotic effect, drugs with a different mechanism of action are available, which can affect blood clotting indirectly in parenteral heparins (e.g., enoxaparin), heparinoid danaparoid sodium, fondaparinux, and in orally active VKAs, such as warfarin, phenprocoumon, and acenocoumarol. Direct inhibition of blood clotting is given by parenteral thrombin-inhibiting hirudin, bivalirudin, argatroban, and in the orally active thrombin inhibitor dabigatran and factor Xa inhibitors, such as apixaban, betrixaban, edoxaban, rivaroxaban [15]. Dabigatran and the factor Xa inhibitors are referred to as direct oral anticoagulants (DOACs), which are prescribed, alone in Germany, in nearly two million patients, mostly over 70 years of age, corresponding to the indications of anticoagulants [16]. In comparison, prescription of VKAs decreased in Germany over recent years and amounts currently to approximately one million patients [16]. The therapy drastically reduces the risk of a fatal heart attack or stroke in vulnerable individuals. However, the antithrombotic effect also increases the risk of bleeding [15]. Since AD involves vascular and hemostatic changes in the pathogenesis, medical repositioning of anticoagulants for treatment of this brain amyloidosis is currently under discussion [6,12,13].

## 3. Alzheimer’s Disease—Toxic Amyloid-β Proteins and Triggered Neuropathogenic Phenomena

The psychiatrist and neuropathologist Alois Alzheimer identified and described protein deposits in the brain tissue of a deceased dementia patient for the first time in 1906 at a conference in Tübingen, Germany [17]. In fact, recent research revealed that a crucial trigger of AD pathogenesis is based on the accumulation of misfolded, toxic Aβ in brain tissue. This theory is currently the primary therapeutic base in trying to stop the course of the disease [18,19,20,21,22,23,24,25,26,27]. Aβ are released from the amyloid-β protein precursor (AβPP) by sequentially acting α-, β- and γ-secretases. AβPP is anchored in the neuronal cell membrane and provides fission products of different length (secreted AβPP). In the healthy brain, Aβ play putative roles in regulation of the hippocampal synaptic function [28], repair of leaks in the blood–brain barrier (BBB) and protection against infections [26]. In AD brain, toxic Aβ are released from AβPP and accumulate in a complex equilibrium of soluble dimers and oligomers, aggregating into insoluble, deposited fibrils (Aβ plaques). Deposition of neuritic plaques of Aβ and oligomeric Aβ are localized between neuron cells in brain parenchyma (in particular Aβ42, Aβ43), while Aβ oligomers of shorter subtype (in particular Aβ40) are deposited around and in the blood vessels of leptomeningeal and cortical arteries and, occasionally, veins. Both events take place particularly in neocortical and hippocampal brain areas. In these specific cerebral regions, excessive neuronal activity and dysfunction and loss of synapses have been observed, strongly correlated with progressive cognitive impairment early in AD [27,29]. Aβ is actively transported into the blood stream through BBB, the vascular interface of the brain [30]. A causal role of these Aβ accumulations in early AD is suggested by the following reasons. Soluble Aβ dimers and oligomers are able to hyperactivate and damage glutamateric neurons and synapses [27,31]. In addition, all major gene modifications, which have been identified so far in association with an increased AD risk, are related to Aβ generation, aggregation and clearance, and microglia responses [4]. Furthermore, the anti-Aβ antibodies aducanumab [32] and donanemab [33] have shown potential to delay cognitive decline by reducing brain Aβ load, when treatment of patients with antibody takes place very early in the disease. These and other anti-Aβ antibodies, such as gantenerumab, BAN2401, are currently under clinical investigation [3,32,33].

Also characteristic of the progressive AD pathogenesis are intraneural deposits of tau protein aggregates, which spread from neuron to neuron and are toxic to them [34]. Under pathological conditions, the microtubule-associated tau protein is modified by phosphorylation and generates insoluble, filamentous tau aggregates, which form neurofibrillary tangles (NFT) typically observed in AD. Filamentous and oligomeric tau aggregates and fragments may contribute to synaptic dysfunction and neuronal cell death, a disorder, known as tauopathies [34]. Aβ boost tau-seeded pathologies in AD, possibly by inducing cyclin-dependent kinase activity for tau hyperphosphorylation and aggregation [35,36]. Both increase in Aβ oligomers and decline in cerebral blood flow (CBF) in AD have been associated with hyperphosphorylation of soluble tau, which relocates from axonal microtubules to dendrites [34,37]. Resulting synaptic dysfunction, together with myelin loss, may lead to cognitive decline [34,37].

Further important pathogenic events, which are also the focus of intensive therapeutic research, include defects in BBB, neural inflammatory processes with reactive oxygen species (ROS) and hydrogen sulfide production, and loss of synapses and neurons [38,39,40,41]. Indeed, elimination of synapses, executed by activated microglia cells, is a known phenomenon in AD [42]. This suggests that microglia are critical to neuronal function and health [42]. When the brain is diseased, microglia cells are activated and their phagocytotic and inflammatory functions are up-regulated [43]. In particular, inflammatory processes, proceeding from activated microglia cells and their release of inflammasome-derived, proinflammatory ASC (apoptosis-associated speck-like protein containing a CARD) protein complexes and cytokine peptides promote the formation and spread of cerebral Aβ deposits [44]. As shown recently, cytokines up-regulate γ-secretase activity for Aβ production through expression of γ-secretase modulatory protein IFITM3 (interferon-induced transmembrane protein 3) in neurons and astrocytes [45]. Accordingly, blocking microglial inflammasome by the inhibitor dapansutrile (OLT1177) reduced microglia activity and cortical Aβ plaque deposition, and rescued cognitive function in AD mouse model [46]. In the early stage of AD, activated microglia cells remove cerebral Aβ deposits. This is in line with their phagocytotic function in the healthy brain, which includes elimination of cellular debris, aggregated proteins and damaged synapses [43]. Accordingly, mutations in the microglial activating gene TREM2, which codes for a receptor protein on the surface of microglia cells, stimulate deposition of Aβ plaques and increase AD risk [47]. Therefore, it is an open question as to which aspects of microglia response on Aβ plaques are promotive of or inhibitory to the disease-causing mechanism [4]. In any case, early processes in AD appear to play a crucial role, because clinical studies have shown that cerebral Aβ accumulate in persons who develop AD 10 to 20 years before cognitive symptoms of the disease are detectable [48]. Similarly, the amount of neurofilament light chain protein (Nfl), which is a bioindicator for neuron cell death, are elevated in the blood of persons with hereditary AD 16 years before the emergence of symptoms [49].

## 4. Alzheimer’s Disease—Cerebral β-Amyloid Angiopathy and Brain Disorder

**Figure 1:**
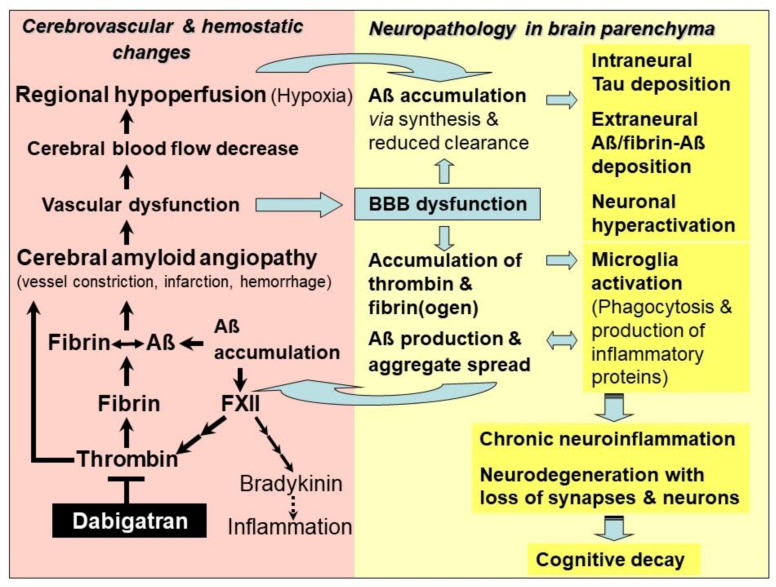
Model of the mechanism of action of the thrombin inhibitor dabigatran for treatment of hemostatic and vascular abnormalities, and derived neurodegenerative changes, contributing to Alzheimer’s disease (AD). As an early and typical event in AD, cerebral blood flow (CBF) decreases and restricts brain perfusion and supply of oxygen (hypoxia) and nutrients, mainly in neocortical and hippocampal areas. Responsible for this hypoperfusion are pathological changes in the cerebrovascular system, which disrupt vascular and blood–brain barrier (BBB) function. This condition is known as cerebral amyloid angiopathy (CAA). In CAA, degradation-resistant fibrin clots containing amyloid-β-proteins (Aβ) are formed (fibrin-Aβ clots) and deposit in and around the walls of blood vessels, causing microvascular constriction, infarction, and hemorrhages. Risk of vessel occlusion is additionally promoted by thrombin-induced platelet aggregation. Aβ intensify this process by activating the blood coagulation factor XII that stimulates the production of inflammatory thrombin and thrombin-mediated formation of fibrin and fibrin-Aβ clots, as well as the synthesis of proinflammatory bradykinin. CAA-induced hypoperfusion (ischemia) and hypoxia up-regulate β- and γ-secretase activities for amplified production of Aβ, oligomers and plaques in nervous system parenchyma. Parenchymal Aβ accumulation is additionally increased by reduced perivascular Aβ clearance, caused by BBB dysfunction. Leakage of BBB also allows plasma proteins, such as thrombin and fibrin(ogen), to pass from the blood vessels into the brain parenchyma, where they accumulate. Thrombin, fibrin and Aβ activate phagocytic microglia cells for production of inflammatory proteins, which further stimulate Aβ production, aggregation and cerebral spread. Fibrin(ogen) interacts with Aβ and promotes fibrin-Aβ clot deposition between neurons. Aβ induce neuronal hyperactivation and synaptic dysfunction, and tau protein-seeded neurotoxic pathologies. Ultimately, this downward spiral of steadily deteriorating vascular and parenchymal changes triggers chronic neuroinflammation, loss of synapses and neurons, and cognitive decay. Dabigatran intervenes at a central point of AD-associated vasculopathies. The drug inactivates free and fibrin-bound thrombin, thus preventing accumulation of inflammatory thrombin, fibrin and fibrin-Aβ clot deposition in cerebral vessels and parenchymal tissue. Consequently, dabigatran could preserve vascular and BBB function, CBF and brain perfusion delivering oxygen and nutrients. Therefore, in brain parenchyma, thrombin-, fibrin- and Aβ-triggered inflammatory milieu and neurodegenerative and cognitive changes could be mitigated or prevented, provided that dabigatran treatment starts immediately after early diagnosis of CAA and AD.

Only in recent years have pathological alterations in cerebral blood vessels, associated with Aβ, received more attention in therapeutic research, although this phenomenon is long-known and typical of AD. Cerebrovascular abnormalities are diagnosed very early in pathogenesis and are referred to as cerebral β-amyloid angiopathy (CAA; Figure 1). Predominantly, neocortical and hippocampal brain areas are affected by CAA, where aggregates of Aβ accumulate and deposit in and around the walls of brain arteries, arterioles and capillaries, interfering with their function [24,41,50,51]. Neuron-derived Aβ are able to migrate to and accumulate in the brain vasculature, far from their site of origin. Resulting vascular dysfunction in Aβ-loaded, diseased brain hampers cerebral blood flow and perfusion, and therefore reduces tissue supply of blood and its constituents, such as oxygen, nutrients (e.g., glucose, ions, amino acids), hormones, proteins and cellular components [22,30,52,53,54]. CAA-damaged blood vessels also obstruct removal of proteins, such as Aβ, from the interstitial brain fluid (ISF) into the blood stream [55,56]. Accordingly, in brains of AD patients, Aβ are deposited first at the periphery of arterioles at the site of supposed interstitial fluid drainage routes [55,56]. Thus, clearance of parenchymal Aβ through distribution and degradation in the blood (perivascular Aβ clearance) is hindered, and Aβ accumulate increasingly in the brain parenchyma [55,57]. In order to investigate the role of CAA-induced vascular dysfunction in AD, transgenic mouse lines are increasingly used as a model, in addition to studies in AD patients. These AD mouse models can exhibit gene modifications affecting Aβ formation, aggregation and local deposition, or in cardiovascular factors, critical for human AD risk [4,58,59]. Recent studies in mouse models and in AD patients revealed that CAA is causally triggered by vascular Aβ deposition, mainly in neocortical and hippocampal brain areas [24,60,61,62,63]. Deposited tau proteins have also been found around Aβ-loaded vessels in cases of sporadic and hereditary CAA [62]. However, this tau deposition is generally not a prominent characteristic of CAA disease [62]. The severity of Aβ deposition in CAA is correlated with an increasing loss of vascular function, decrease in CBF, hypoperfusion and deficient brain supply of oxygen (hypoxia) and nutrients [22,63,64] (Figure 1). Vascular contribution to AD has been exemplarily demonstrated by studies using living human brain biopsy tissue and in vivo AD mouse model, as well as in mechanism studies, by adding Aβ and drugs to capillaries in rat cortical slices [64]. Close correlation has been found between Aβ deposition in cortical capillaries and increasing contraction of their pericytes on the outer vessel walls [64]. In contrast, arterioles and venules stayed unchanged. The Aβ-mediated mechanism for pericyte contraction appears to be based on ROS-induced endothelin-1 release [64]. Contraction of pericytes reduced the diameter of capillaries, which led to capillary constriction (vasoconstriction) and decreased blood flow in the vessels. The result was a chronic hypoperfusion (ischemia) of affected brain areas, leading to hypoxia, a well-known phenomenon in early AD [64]. Vascular dysfunction and concomitant decrease in CBF, monitored in the range of 25%, have been observed in AD patients [53,54,55], as well as in AD mouse model [37,63]. Studies have also shown that brain hypoxia/ischemia conditions induce synthesis of Aβ from the neural precursor AβPP via up-regulation of gene expression for β-secretase1 (BACE1) and activation of γ-secretase, which occurs practically in a self-amplifying process [65,66,67]. This pushes inflammatory and degenerative changes, leading to cognitive decline [66,67]. Clinical observations further revealed that CAA and AD pathology co-occur in the brain of AD patients with an incidence of 82%–98% [68]. Therefore, restriction of vascular function in CAA and resulting CBF decrease and hypoperfusion are considered to be an early and critical pathophysiological mechanism in AD [50,65,69], which takes its origin from cerebrovascular Aβ deposition and evoked vasculopathies. These include, besides Aβ-induced vessel constriction, microinfarction (occlusion) and micro-hemorrhagic (bleeding) events, which injure BBB and cause inflammatory and degenerative alterations in brain tissue [24,69,70] (Figure 1). Interestingly, physical exercise, which improves CBF and brain perfusion, has been found to slow down neuropathological processes in persons with genetic AD predisposition [71], as well as in AD mouse model [72].

## 5. Alzheimer’s Disease—The Role of Thrombin, Fibrin and Amyloid-β-Containing Fibrin Clots

Particularly in the last decade, studies have evidenced that accumulation of toxic Aβ, thrombin and fibrin/fibrinogen co-occur in brains of genetic and sporadic AD patients and in AD mouse brain [41,50,73,74,75,76,77]. It has been found that fibrin(ogen) colonizes together with Aβ along cerebral vessel walls and in the nervous system parenchyma [50,73,77]. Thrombin and fibrin(ogen) trigger neuroinflammatory processes and, furthermore, fibrin(ogen) is able to interact with Aβ [50,73,75,76,77] (Figure 1). In parenchymal tissue, accumulation of fibrin(ogen) and thrombin is caused by the circumstance that BBB is increasingly leaky for plasma proteins, due to the evolving vascular dysfunction in CAA [70,78,79]. Fibrinogen can transgress from the blood vessels into the parenchymal tissue. Here, it is converted into fibrin by thrombin and additional perivascular tissue factor and procoagulant proteins, which are also abundant in this tissue after vascular damage [70,78,79]. Leakage of BBB results from hemorrhage and infarction injuries, and from degeneration of pericytes in capillary walls, which interrupts junctions between adjacent endothelial cells [79]. Overall, BBB dysfunction and resulting processes are associated with increased Aβ production, microglia activation, and malfunctioning and loss of synapses and neurons [30] (Figure 1).

Interestingly, recent studies have shown that fibrin(ogen) is able to bind to Aβ via its central region, while Aβ binds the αC region of fibrinogen (based on Aβ42) [74,76]. Interaction of fibrin(ogen) with Aβ promotes Aβ oligomerization and forms fibrin clots, which contain Aβ (fibrin-Aβ clots) [73,76,80]. These fibrin-Aβ clots exhibit an abnormal structure of the fibrin mesh, making them more resistant against the degrading enzymes in plasmin fibrinolysis than normal clots [73,76,80]. It has been found that these Aβ-containing fibrin clots deposit in and around cerebral blood vessels of neocortical and hippocampal brain areas of CAA. They also deposit, together with Aβ oligomers and plaques, in parenchymal brain areas of dystrophic neurites [73,74,76,77,78]. In blood vessels, fibrin-Aβ clot deposition leads to disruption of vascular and BBB function, and inhibits blood flow in areas of CAA [50]. In parenchymal brain tissue, fibrin-Aβ clot deposition is associated with neuroinflammatory and degenerative processes, leading to the death of synapses and neurons [50]. In patients with hereditary CAA, mutations in Aβ were identified, which promote formation and deposition of cerebral fibrin-Aβ clots in AD pathogenesis [80]. On the other hand, chemical inhibitors of fibrin(ogen)-Aβ interaction, such as RU-505, which bind directly to Aβ, are able to prevent the formation of fibrin-Aβ clots [50,81]. Accordingly, long-term treatment with RU-505 in AD mouse model significantly reduced vascular amyloid deposition and vessel occlusion, as well as cerebral microgliosis and cognitive impairment [50,81]. Interestingly, the pharmacophore for thrombin binding of the DOAC dabigatran [14] exhibits certain structural similarity to the amide side chain part of RU-505 [80], which is possibly involved in fibrin(ogen)-Aβ interaction (Grossmann, K.; personal comment). It might be worth investigating whether dabigatran is able to interfere with fibrin-Aβ clot formation, in addition to its known thrombin-inhibiting activity (Grossmann, K.; personal comment).

As shown in AD patients and mouse models, Aβ is also able to interact with different coagulation factors, thus promoting a prothrombotic and proinflammatory milieu [50]. Particularly, the blood clotting factor FXII in the cascade for thrombin production is activated by Aβ (Figure 1), leading to an increased production of fibrin and fibrin-Aβ deposits in brain vessels and parenchymal tissue [82,83]. Concomitantly, rising amounts of inflammatory thrombin and its precursor prothrombin were found in cerebral micro-vessels, collected from AD patients, and in parenchymal tissue, particularly in neurons, glial cells and intraneural tau deposits [83,84,85,86,87]. Besides their function in hemostasis, thrombin and fibrin are recognized as key mediators of a multitude of inflammatory processes in the vessel walls and parenchymal tissue. These include direct activation of microglia cells and astrocytes, and triggering of neuronal damage in the AD brain [75,78,86] (Figure 1). Thrombin is a pleiotropic enzyme that also evokes, directly and indirectly, a plethora of non-hemostatic, cellular effects in multiple tissue types through interaction with protease-activated receptor (PAR) signaling, and has been implicated in a variety of diseases [88]. Moreover, in response to vascular injury, thrombin induces platelet aggregation, which can further promote vessel occlusion in CAA [75]. Parenchymal fibrin activates microglia cells by binding of the cryptic fibrin epitope γ377–395 with the microglial receptor complement receptor 3 (CR3), which boosts inflammatory processes [89,90]. On the other hand, blocking of this fibrin epitope by antibody lowered the inflammatory and degenerative milieu in AD mouse brain [89]. Cerebral inflammation is also stimulated by plasmin and plasminogen of the hemostatic system [91]. Furthermore, studies have shown that activation of the blood clotting factor FXII by Aβ induces synthesis of the proinflammatory and vasoactive nonapeptide bradykinin, via the synthesis steps plasma prekallikrein and high molecular weight kininogen [83,92] (Figure 1). In AD patients, increasing plasma bradykinin levels correlate with the severity of cognitive impairment [93]. As shown in AD mouse model, Aβ-induced FXII activation and brain pathology and cognitive impairment appear to be causally linked [94].

To sum up, recent results confirm a key role of Aβ, thrombin and fibrin in CAA and AD pathogenesis. The cerebrovascular system and BBB are impaired in their function, regional CBF and brain perfusion decrease, and tissue supply of oxygen and nutrients arrives at a limit (Figure 1). The result is a downward spiral of ever-worsening and amplifying vascular, neuroinflammatory and neurodegenerative changes. In brain parenchyma, this disaster is further intensified by hypoxia-induced production of Aβ and fibrin-Aβ deposits, and accumulation of thrombin, fibrin(ogen) and Aβ, which is additionally amplified through reduced perivascular clearance. Originally, these effects have their cause in CAA and trigger vascular and BBB dysfunction, and in Aβ-induced FXII activation in the hemostatic system. Further detrimental effects include Aβ-intensified tau and neuronal pathology, and the activation of phagocytic microglia by Aβ, thrombin and fibrin. Microglia cells produce inflammatory proteins after activation, which trigger further Aβ production and cerebral spread of aggregates, as well as chronic neuroinflammation. Ultimately, these processes lead to the death of synapses and neurons, and cognitive decay (Figure 1).

## 6. Alzheimer’s Disease—Treatment with DOAC-Type Anticoagulants

The new therapeutic approach with DOAC-type anticoagulants relies on their specific mechanism, which acts on key hemostatic drivers in CAA and related neuronal defects in AD. By inhibiting activity or synthesis of thrombin, DOACs are capable of blocking inflammatory thrombin and fibrin for microglia activation, and inhibiting formation of degradation-resistant Aβ-containing fibrin clots. Fibrin-Aβ clots accumulate in the nervous system parenchyma, leading to neuronal damage [73,77]. They also deposit in blood vessels in areas of CAA, where they cause vascular dysfunction, leading to the collapse of BBB, CBF and brain perfusion [41,50]. DOAC treatment has the potential to counteract CAA and vascular dysfunction, and to maintain brain perfusion and supply of oxygen and nutrients. CAA-resulting parenchymal changes, which include accumulation of Aβ, thrombin and fibrin, tauopathies, microglia activation, neuroinflammation, loss of synapses and neurons, and cognitive decline, could also be mitigated or prevented (Figure 1). In order to achieve the best success, AD patients should be treated with DOAC immediately after early diagnosis of vascularly-driven neurodegenerative changes in AD.

First preclinical studies in AD mouse model, treated with the parenteral heparin-type anticoagulant enoxaparin, revealed that indirect thrombin inhibition is able to reduce cortical Aβ deposition [95,96]. In addition, application of the orally active direct thrombin inhibitor dabigatran lowered levels of vascular inflammatory proteins and ROS formation [86], and microglia activation [97]. It has been proposed that thrombin inhibitors might be able to alleviate symptoms in AD, due to their dampening impact on vascular proinflammatory thrombin [75,98]. In line with the hypothesis of a beneficial effect of anticoagulants on AD pathogenesis [12,13,75,98], recent results from a preclinical study in AD mouse model showed the potential therapeutic usefulness of DOAC medication against AD [99]. As reported by Cortes-Canteli et al., long-term treatment with dabigatran prevents cerebral fibrin clot deposition, CBF decrease, hypoperfusion, and memory decline [99]. Concomitantly, dabigatran treatment drastically reduced accumulation of Aβ plaques and oligomers and neuroinflammatory activity, evaluated by occurrence of phagocytic microglia and infiltrated T cells. Moreover, BBB function appeared to be intact, inferred from non-existent astrogliosis and pericyte alterations, and incidents of intracerebral hemorrhages were not observed [99].

For possible daily, permanent treatment of vascular dysfunction in AD patients, orally active anticoagulants have advantageous handling and mechanisms of action, relative to heparin infusion therapies. Parenteral heparin applications are usually prescribed in acute situations for a short period of time. Besides their handling, they have additional disadvantages, which include that fibrin-bound thrombin, a trigger of fibrin clot formation, is not being inactivated, and unspecific binding of plasma proteins unpredictably interferes with the anticoagulative effect [14,100]. In addition, the risk of thrombocytopenia increases [14,100]. Alternatively, natural thrombin-inhibiting hirudin was applied to patients with mild-to-moderate AD in an open-labeled study [101]. The result revealed that hirudin plus the cholinesterase inhibitor donepezil slow down the rate of cognitive decline, compared to donepezil alone [101]. Although hirudin has advantages over heparins, such as no direct effects on platelets and inactivation of fibrin-bound thrombin, in therapeutic use of hirudin and its derivatives an increased risk of bleeding complications has been observed [102].

Orally active anticoagulants comprise the familiar VKA-type anticoagulants, prescribe now for decades, such as warfarin (Coumadin^®^), phenprocoumon (Marcumar^®^, Falithrom^®^), and acenocoumarol (Sintrom^®^). However, for treatment of AD patients, VKA-type anticoagulants exhibit undesirable side effects, which include bleeding complications (inclusive slow-acting reversal effect of vitamin K antidote), coumarin necrosis, vitamin K deficiency effects on important proteins of the vascular and nervous system (e.g., vitamin K-dependent proteins Gas6, MGP; key enzymes of sphingolipid synthesis), as well as dietary and pharmacodynamic interactions [14,103,104]. In this respect, the novel, specific-acting DOAC-type anticoagulants, which have been approved for therapeutic use and introduced into the medicine market over the last decade [14], are more suitable [12,14]. These include the blood clotting factor Xa-inhibitors apixaban (Eliquis^®^), betrixaban (Bevyxxa^®^), edoxaban (Lixiana^®^), rivaroxaban (Xarelto^®^), and the blood clotting factor IIa (thrombin)-inhibitor dabigatran (orally administered prodrug: dabigatran etexilate, Pradaxa^®^) [14].

Among the DOACs, dabigatran could be favored for treatment of vascular and hemostatic changes contributing to AD, based on the following rationales [12,13]. Dabigatran is the only direct thrombin inhibitor, available in the class of new antithrombotic DOACs [105]. Dabigatran is approved for several indications, such as prevention of stroke in patients with nonvalvular atrial fibrillation and prevention of venous thromboembolism in orthopedic surgery [14,105]. Dabigatran etexilate is the peroral prodrug form, which releases in vivo by unspecific esterase activity the active ingredient dabigatran. Dabigatran binds, specifically and directly, to free soluble and fibrin(clot)-bound thrombin in the blood, and inhibits, reversibly and competitively, the enzymatic activity of thrombin for fibrin formation and fiber networking. In addition, dabigatran blocks soluble thrombin, which can induce platelet aggregation and inflammatory and neurotoxic effects in the brain [14,75,88,105]. For AD therapy, this unique feature of dabigatran is particularly beneficial, compared to VKAs and factor Xa-inhibiting DOACs. The latter two types solely prevent the production of thrombin [14]. Furthermore, in comparison with the VKA warfarin, dabigatran has a shorter half-life and minimal drug–drug interactions observed in treated humans [14,104,105]. In addition, dabigatran showed a lower risk of ischemic stroke, intracranial hemorrhage and mortality in elderly patients with atrial fibrillation, but dose-dependently, an increased risk of gastrointestinal bleeding [106,107]. According to a retrospective observer study in approximately 130,000 patients over two years by the U.S. Food and Drug Administration (FDA), an incidence rate for harmful intracranial hemorrhage per 1000 person-years of 3.3 was observed after dabigatran treatment, whereas a rate of 9.6 was found in the case of warfarin [107]. Recently, this reduced risk of intracranial bleeding in DOAC treatments, versus warfarin, could also be confirmed in a new-user retrospective cohort study using people with atrial fibrillation and dementia [108]. In addition, when compared with the factor Xa-inhibitor rivaroxaban in a study with elderly patients with atrial fibrillation, dabigatran treatment was associated with significant decreases in intracranial hemorrhage and gastrointestinal bleeding [109]. Accordingly, in mouse models of AD and CAA, no increase in intracerebral hemorrhage or frequency of acute microhemorrhage has been observed after dabigatran treatment [110,111]. Overall, these studies predict a lower cortical bleeding risk, compared to VKAs, if dabigatran was used in vulnerable AD patients. An additional favorable property of dabigatran is a potent anti-inflammatory effect, which was demonstrated in a mouse model of fibrosis [112]. Due to its mechanism of action, dabigatran exhibits no vitamin K deficiency effects and no food interactions with its anticoagulative activity [105]. Therefore, dabigatran has a predictable pharmacokinetic profile for effective anticoagulation [105], which can be abolished within minutes by treatment with the dabigatran-specific antibody antidote idarucizumab (Praxbind^®^) [113]. This means that a bleeding phenomenon can be stopped immediately after diagnosis [14,113]. Fast-acting antidotes have also recently been available for factor Xa-inhibiting DOACs [14]. Nevertheless, long-term anticoagulation in the mostly aging and comorbid AD population, which are more likely to bleed due to fragility of blood vessels in advanced stage of CAA, must be examined very carefully for the risk of bleeding [114]. Here, an individually tailored program with dabigatran could possibly be favourable.

## 7. Alzheimer’s Disease and Other Brain Amyloidosis—Outlook for Therapeutic Use of Dabigatran

As discussed above, DOACs can achieve beneficial effects on vascular and neural dysfunction, contributing to AD. Particularly, the direct thrombin inhibitor dabigatran opens up the opportunity to effectively combat both the accumulation and harmful effect of inflammatory thrombin and fibrin, as well as the production and deposition of Aβ and fibrin-Aβ clots in cerebral blood vessels and parenchyma. Consequently, dabigatran could be able to treat CAA and related vascular dysfunction, in order to mitigate or prevent resulting effects, such as BBB dysfunction, CBF decrease, brain hypoperfusion and lack of oxygen and nutrients, as well as progressive neuroinflammatory, degenerative and cognitive changes (Figure 1).

In accordance, a retrospective registry study in Sweden (2006–2014) on more than 400,000 patients with atrial fibrillation yielded a considerably lower risk of dementia after treatment with DOACs or the VKA warfarin [115]. Likewise, association between oral anticoagulant use and a lower risk of dementia was demonstrated in a retrospective cohort study (2000–2017; based on UK primary care data) in participants with newly diagnosed atrial fibrillation without incidence of dementia [116]. Furthermore, a retrospective clinical study (2010–2014) was carried out in more than 5000 elderly patients with atrial fibrillation, who received long-term anticoagulation with DOACs (apixaban, dabigatran, rivaroxaban), in comparison to warfarin [117]. The results revealed that DOACs have a better long-term efficacy and safety in prevention of thromboembolism, bleeding and death, associated with a lower risk of cerebral ischemic events and new-onset dementia [117]. Summarizing all previously reported studies in patients with atrial fibrillation and oral anticoagulation, a meta-analysis and systematic review came to the conclusion that DOAC treatment protects against dementia [118]. Currently, a clinical observer study is underway, which has incident dementia as primary endpoint in patients with atrial fibrillation and treatment with dabigatran versus warfarin [119].

In conclusion, there are strong rationales that would warrant realization of a first pilot clinical study using dabigatran against AD [12,13,41,75]. These rationales result from (1) recent research on the roles of thrombin, fibrin and Aβ in triggering vascular and neurological changes in AD [41,50,75,78], (2) preclinical studies showing beneficial effects of dabigatran treatments [86,97,99,112] and (3) clinical observer studies [115,116,117,118]. For such a clinical pilot study, the oral direct thrombin inhibitor dabigatran with its well-known application and safety profile, including availability of a fast-acting antidote [105,113], would be a reasonable drug solution to investigate the therapeutic value of thrombin inhibition in AD. This intervention study should ideally be carried out in close cooperation between neurological and cardiovascular medicine. In this way, dabigatran effects on the early course of CAA and neuropathy in AD patients could be studied comprehensively and competently. The possible method repertoire used could include position emission tomography (PET) and magnetic resonance imaging (MRI) technologies for visualizing brain biomarkers, such as Aβ deposits, bleeding events, and CBF dynamics [22,114,120,121]. Invasive cerebrospinal-fluid (CSF) analyses, e.g., on Aβ, tau [121], and preferentially, blood biomarker tests, which indicate neurodegenerative (e.g., Nfl [49], Aβ fragments [121]) and hemostatic alterations (e.g., thrombin, fibrin clot forming), as well as cognitive ability checks, can supplement the study. However, cost/benefit of the intended methods should be parsed before the analysis [122]. Highly important for this clinical study is that participating persons are carefully evaluated, particularly according to their bleeding risk, using, e.g., clinical HAS-BLED-Score criteria and imaging methods [13,14]. As with any drug, benefit as well as risk of undesirable effects must be individually weighted and discussed, as the basis for consent of participants. It is also extremely critical for the success of the therapy with dabigatran that subjects, who are at the beginning of AD pathogenesis, are selected according to a lesson learned from past clinical studies on Aβ-targeting therapies [122]. At best, the onset of AD should be diagnosed very early, using, e.g., AD blood biomarkers and confirmation by CSF analysis and brain imaging methods, if cognitive failures are already suspected or feared. Then, after positive diagnosis, treatment with dabigatran should start immediately, in order to possibly combine the highest therapeutical efficiency with the lowest risk of bleeding. In the case of individuals with genetically high AD risk predisposition (early onset AD), dabigatran could/should be administered prophylactically, before this terrible disease begins.

For implementation of a clinical study on dabigatran treatment against AD, it would be desirable that appropriate clinical institutes are motivated and supported by funding associations and pharmaceutical companies. Ultimately, positive results could be aimed to expand dabigatran approval to repositioning for AD [6,123]. For this well-known medicament, therapeutic translation might be faster and less expensive to achieve. An off-label-use might also be considered in individual cases outside existing approval [123]. Interestingly, a first clinical phase I double-blind intervention study has been recently announced for patients in the early stage of AD [124]. Dabigatran is envisaged for this therapeutic approach. Nearly 80 years ago, VKA-type anticoagulants, beginning from the naturally occurring dicumarol, were introduced to clinical medicine [11]. At this time, these drugs gained particularly fame and impetus for cardiovascular use, after the treatment of US President Eisenhower’s heart attack in 1955 [11]. Currently, a similar prominent key event might be needed, to overcome concerns and hesitant waiting, and the rationales discussed in this review may possibly accelerate matters in order to carry out detailed clinical studies of dabigatran use against AD.

Moreover, the question arises as to whether toxic proteins and hemostatic factors also trigger microvascular, BBB and CBF alterations contributing to neurodegenerative disorders in other brain amyloidosis, such as Parkinson’s disease. Interestingly, a recent study revealed that overexpression of human α-synuclein, the toxic counterpart to Aβ in Parkinson’s disease [23], led to brain vascular pathology, BBB dysfunction with fibrinogen leakage, and pathological activation of pericytes in a mouse model of this disease [125]. Possibly, further investigations on the role of brain vasculature [126] and hemostatic factors, e.g., thrombin, fibrin(ogen) [78], contributing to neurodegeneration in other amyloidosis, such as Parkinson’s disease, Huntington’s disease, and amyotrophic lateral sclerosis, might lead to the hypothesis that DOACs, such as dabigatran, could be helpful also in treating vascular dysfunction in these neurodegenerative diseases.

## Abbreviations

Aβ	amyloid-β proteins
AβPP	amyloid-β protein precursor
AD	Alzheimer’s disease
BBB	blood-brain barrier
CAA	cerebral amyloid angiopathy
CBF	cerebral blood flow
CSF	cerebrospinal-fluid
DOAC	direct oral anticoagulant
FXII	blood clotting factor XII
Nfl	neurofilament light chain protein
ROS	reactive oxygen species
VKA	vitamin K antagonist
Xa	blood clotting factor Xa
